# Still's Disease and Myopericarditis

**DOI:** 10.7759/cureus.4900

**Published:** 2019-06-14

**Authors:** Manish Kumar, Varun Tandon, Nerea Lopetegui Lia, Shriyanka Jain

**Affiliations:** 1 Internal Medicine, University of Connecticut Health Center, Farmington, USA; 2 Internal Medicine, University of Arizona College of Medicine - Phoenix, Phoenix, USA; 3 Rheumatology, University of Connecticut Health Center, Farmington, USA

**Keywords:** myopericarditis, myocarditis, adult onset still's disease

## Abstract

Adult-onset Still’s disease (AOSD) has a vast array of clinical presentations. Myopericarditis is one of the rarest cardiopulmonary manifestations of the disease and due to its rarity, the literature on the association of myocarditis with AOSD is sparse. Herein, we describe an interesting case of a 44-year-old male who presented with chest pain following exertion. He was febrile at the time of presentation and exam revealed inflammation in various joints. Electrocardiogram showed diffuse ST segment elevations in the precordial leads. Laboratory results revealed elevated troponin of 3.17 (<0.05 ng/mL) and CK-MB of 6 ng/mL along with elevated ferritin of 6225 (16-336 ng/mL). Cardiac MRI showed early and late gadolinium enhancement consistent with myocarditis. The patient was started on steroids and non-steroidal anti-inflammatory drugs (NSAID) resulting in clinical improvement. This case highlights the critical importance of diagnosis of pericarditis and myocarditis in patients with AOSD, as a missed diagnosis can lead to significant morbidity and mortality.

## Introduction

Adult-onset Still’s disease (AOSD) has a wide-ranging presentation and is considered a diagnosis of exclusion. The most common cardiopulmonary manifestations of AOSD include pericarditis, pleural effusions, and transient pulmonary infiltrates [1]. Myocarditis is one of the rarest presentations. Herein, we describe an interesting clinical scenario of a patient with AOSD who presented with chest pain due to acute myopericarditis.

## Case presentation

A 44-year-old male presented to the hospital with chest pain associated with generalized malaise and arthralgia. The patient started having constant, mild to moderate, substernal heaviness while jogging. The pain was radiating to right shoulder and back, relieved by leaning forward and worsened by deep inspiration. He had joint pain with swelling in his knees, elbows, and wrists. He complained of low-grade fevers for the last two to three days prior to presentation. A complete review of other systems was unremarkable. He denied a history of recent upper respiratory tract infection, tick bite, or skin rash. His past medical and surgical history included juvenile rheumatoid arthritis, hypertension, and bilateral hip and shoulder replacement. His only medication was as needed ibuprofen.

**Figure 1:**
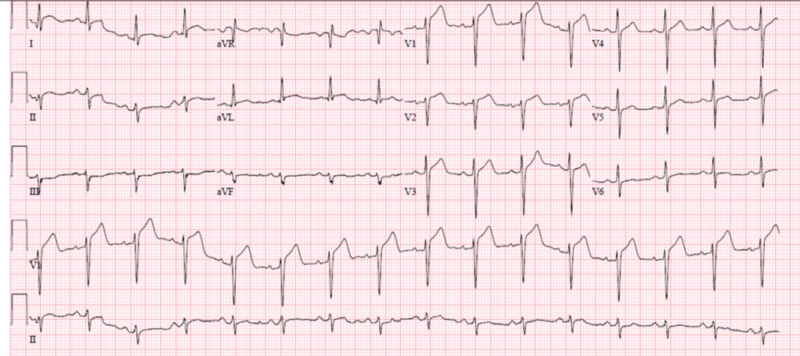
EKG at the time of arrival showed ST-segment elevations in precordial leads EKG, electrocardiogram

In the emergency department, he had a temperature of 102.5 °F, heart rate of 99 beats per minute with other vitals within normal limits. He was in mild distress due to chest pain. The cardiopulmonary examination was unremarkable. Musculoskeletal examination revealed mildly swollen left knee with local tenderness and restricted range of motion (ROM). He had bilateral wrist and elbow flexion contracture with limited ROM. Electrocardiogram showed diffuse ST segment elevations in the precordial leads (Figure 1). Laboratory work was remarkable for a white count of 11.4 (4.0-10.5 k/uL). Cardiac enzymes showed serum troponin levels of 3.17 (<0.05 ng/mL). His ferritin levels were elevated to 6225 (16-336 ng/mL). The basic metabolic profile was within normal limits. Given EKG changes and elevated troponins, a bedside echocardiogram was performed that showed global left ventricular hypokinesia with an ejection fraction of 50% to 55% and no pericardial effusion.

**Figure 2:**
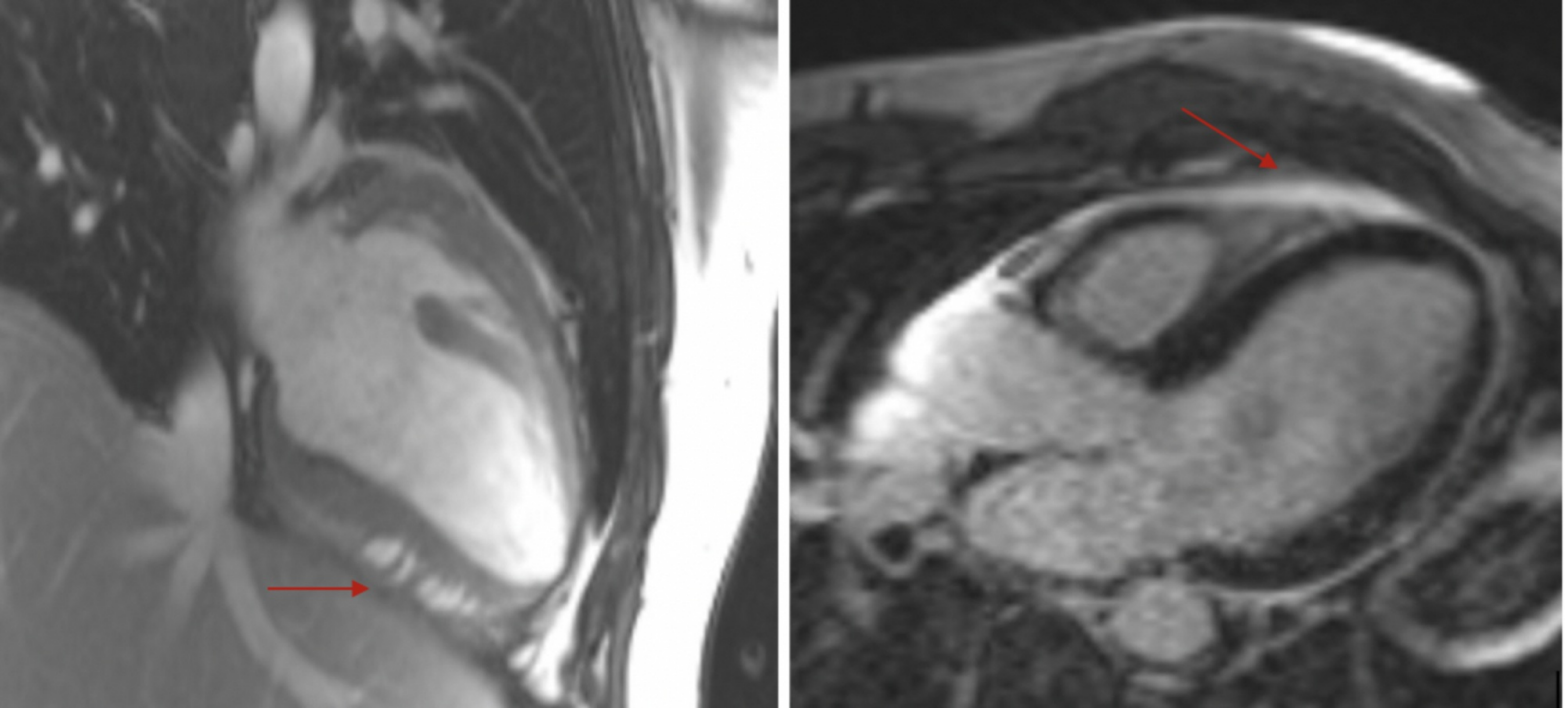
Cardiac MRI showing early (left arrow) and late (right arrow) gadolinium enhancement consistent with myocarditis MRI, magnetic resonance imaging

Given the diffuse ST-segment elevations as well as elevated cardiac enzymes, there was a strong suspicion of myocarditis and cardiac ischemia. The patient was febrile at the time of presentation and along with elevated troponin and ferritin (acute phase reactant and also due to Still's disease), symptoms were attributed to probable myopericarditis. Cardiac catheterization was not done. Cardiac MRI was used to help make the diagnosis without the need for invasive cardiac biopsy. It showed early and late gadolinium enhancement consistent with myocarditis (Figure 2). He was started on intravenous steroids resulting in improvement in the chest and joint pain. His troponin levels trended down to 0.78 ng/mL and white count returned to normal. The patient was eventually discharged from the hospital on prolonged steroid taper. His chest pain resolved; however, he continued to have joint symptoms following which he was started on Anakinra (IL-1 receptor blocker) as an outpatient.

## Discussion

The presentation of adult-onset Still’s disease (AOSD) is varied and is usually diagnosed using Yamaguchi criterion. The diagnosis is clinical and not based upon serology. The most common cardiopulmonary manifestations of AOSD include pericarditis, pleural effusions, and transient pulmonary infiltrates [1]. Because of serosal involvement, cardiac manifestations in AOSD are usually in the form of pericarditis [1]. Despite being a life-threatening condition, the literature on the association of myocarditis with AOSD remains sparse. Its prevalence is rarely reported even in the largest case series [2-3].

The diagnosis of pericarditis and myocarditis in AOSD patients remains critical, as a missed diagnosis can lead to significant disease. Patients with AOSD and myocarditis (AOSD+M) tend to be younger, more often male, and are more likely to have pericarditis with higher WBC counts and higher serum ferritin levels [3]. Some case reports have reported the occurrence of pericarditis with cardiac tamponade and myocarditis with heart failure in patients with AOSD [4-5]. 

Myocarditis is suspected when there is clinical evidence of heart failure along with acute enlargement of the heart on chest X-ray and echocardiographic findings of dilated chambers with decreased ventricular function. The diagnosis of myocarditis can be confirmed with a transvenous cardiac biopsy. Recently, cardiac MRI is being more favored for the diagnosis of myocarditis. A combination of T2-weighted MRI and post-gadolinium early and late T1-weighted MRI has shown good sensitivity (67%) and specificity (91%) in diagnosing myocarditis [6]. There are new novel approaches for characterizing tissue including combining imaging criteria with serum markers to further increase the utility of cardiac MRI in the future [6]. 

The treatment of acute myocarditis in AOSD (AOSD+M) is not different from the treatment of acute myocarditis in the general population. Steroids are the first-line treatment for AOSD+M patients and have been shown to be effective and sufficient for treatment [2]. Therefore, steroids should not be delayed if there is a high probability of the disease. In relapsing and resistant cases, intravenous immunoglobulin, methotrexate, and anti-TNF alpha agents such as etanercept have been used successfully [3,7]. NSAIDs are usually avoided as they may impair the myocardial healing. Some patients may develop systolic dysfunction and require neurohormonal blockade with an ACE-I or ARB, a β-adrenergic blocker, and diuretics. Patients should have a long-term follow-up with repeat echocardiography and should and refrain from intense physical activity until ventricular recovery has been documented by noninvasive imaging [8].

## Conclusions

AOSD usually has serosal cardiopulmonary manifestations such as pericardial inflammation and cardiac tamponade. Pericarditis and myocarditis are not very common occurrences in these patients. However, clinicians should be aware of the critical importance of diagnosis as these conditions can be life-threatening. Patients usually improve with steroids and NSAIDs are generally avoided.
